# Students’ perspectives of undergraduate research methods education at three public medical schools in Uganda

**DOI:** 10.11604/pamj.2016.24.74.9410

**Published:** 2016-05-24

**Authors:** Ian Guyton Munabi, William Buwembo, Ruberwa Joseph, Kawungezi Peter, Francis Bajunirwe, Erisa Sabakaki Mwaka

**Affiliations:** 1 Department of Human Anatomy, School of Biomedical Sciences, Makerere University College of Health Sciences, Kampala, Uganda; 2 Medical student, Makerere University College of Health Sciences, Uganda; 3 Mbarara University of Science and Technology, Mbarara, Uganda

**Keywords:** Research methods, statistical education, undergraduate, health professional

## Abstract

**Introduction:**

In this study we used a model of adult learning to explore undergraduate students’ views on how to improve the teaching of research methods and biostatistics.

**Methods:**

This was a secondary analysis of survey data of 600 undergraduate students from three medical schools in Uganda. The analysis looked at student's responses to an open ended section of a questionnaire on their views on undergraduate teaching of research methods and biostatistics. Qualitative phenomenological data analysis was done with a bias towards principles of adult learning.

**Results:**

Students appreciated the importance of learning research methods and biostatistics as a way of understanding research problems; appropriately interpreting statistical concepts during their training and post-qualification practice; and translating the knowledge acquired. Stressful teaching environment and inadequate educational resource materials were identified as impediments to effective learning. Suggestions for improved learning included: early and continuous exposure to the course; more active and practical approach to teaching; and a need for mentorship.

**Conclusion:**

The current methods of teaching research methods and biostatistics leave most of the students in the dissonance phase of learning resulting in none or poor student engagement that results in a failure to comprehend and/or appreciate the principles governing the use of different research methods.

## Introduction

Research is globally recognised as a major output of universities and other institutions of higher education. On the other hand the teaching of research methods has received a limited amount of attention [1, 2]. Also, health professional training curricula have increasingly become congested leading to the allocation of inadequate time to hands-on teaching of research methods and related research statistical approaches [3]. Much of the university teaching on research methods focuses more on knowledge transfer, while giving little or no attention to context and application of the acquired knowledge [2, 4]. This lack of context makes the learning of research methods and biostatistics even more difficult for the research naïve undergraduate health professional students. There is thus a need to look at the teaching of research methods, since equipping undergraduate students with these research literacy skills leads to increased research output by the individual [5], and eventual prominence of the university. As one of the consumers of information generated in institutions of higher education, students are a valuable source of feedback leading to quality improvements [6, 7]. Concerns about quality are driven in part by globalisation of higher education, the increasing student numbers and calls by governments for accountability [6]. Students are also a more immediate constituency, who need to be listened to for effective management of their expectations and preferences [8]. These expectations do change over time [9], and are influenced by: culture [10]; gender [11]; age; university type [12] and mode of study [13]. Also students require an emotionally supportive atmosphere where they can explore, conjecture, hypothesize, and brain storm [4]. Since students’ research outputs count greatly to research related status of most institutions of higher education, then keeping track of students research related expectations and performance is a key quality control measure. This in turn will impinge on how institutions teach research methods. It is well known that assessment drives learning, by getting students to focus on information that can help them pass exams, or that they have come to believe will be of importance in future clinical practice. Yilmaz gives three specific competencies required to apply statistics: the ability to link statistics to real-world situations; knowledge of basic statistical concepts; and the ability to analyse and disseminate research results in a clear manner [14]. Cognitive and constructivist theories of learning stress that it is important to understand the learning process from the students’ perspective [15]. In this study we explored undergraduate students’ views on how to improve the teaching of research methods and biostatistics at three public universities in Uganda.

## Methods

This was a secondary analysis of undergraduate student's survey data from three purposively selected public medical schools in Uganda namely: Makerere University College of Health Sciences (MakCHS), Gulu University (GU) and Mbarara University of Science and Technology (MUST) [16]. All these universities have periods for community placement to allow students to learn in real life settings as described in their newly revised competence based curricula. In GU and MUST the programs related to teaching of research methods and statistics are delivered using a didactic approach while MakCHS predominantly uses a problem based learning approach. The survey had 600 undergraduate student respondents on various health professional degree programs that included: Nursing, Pharmacy, Cytotechnology, Medical radiography, Dentistry, Medicine and surgery. The analysis looked at students’ responses to an open ended section of a questionnaire on their views on the undergraduate teaching of research methods and statistics: “*Please suggest any ways in which your undergraduate teaching in research methods and statistics could have been more useful?*”. It is important to note that the pharmacy and nursing programs have a research project as a requirement for the award of the degree at all the three universities. The students’ responses were previously captured verbatim in Epidata and then exported to Microsoft word for editing before being saved as a single text file for analysis. The responses were then subjected to qualitative phenomenological data analysis informed by the model of adult learning proposed by Taylor and Hamdy (2013) [17], using a text based open source RQDA package in the R statistical analysis software [18, 19]. The responses were read several times to identify codes from which themes were later deduced. Memos and annotations were used to capture additional observations for each item with respect to the researchers’ experiences as a practicing health professional. Reading and coding of the responses was repeated several times till no new codes or themes were identified. This analysis was done by two of the researchers independently with a third researcher being called in to resolve any conflicts arising during analysis. The coded files were shown to two students as an additional authentication, member-checking step prior to writing up this paper. Additional quantitative analysis was done using STATA 12 by running the various codes against other variables in the data set to obtain additional inferential statistics using the one-way ANOVA with the level of significance set at 0.05. Ethical approval was obtained from Makerere University School of Biomedical Sciences Research and Ethics Committee (IRB number SBS-045) and registered with Uganda National Council of Science and Technology. Return of both the dully completed informed consent form and questionnaire were taken to indicate the students consent to participate in the study. No personal identifier marks were captured or used at any part of the analysis. Students received a drink and small snack equivalent to 1 US dollar as refreshment for participating in the study. Refusal to participate in the study did not in any way affect the student's access to materials or services they were entitled to.

## Results

There were 600 respondents to this survey of whom 516 responded to the open-ended question. Table 1 provides a summary of the relative frequencies observed for each coded item from each of the three participating institutions (Table 1). No differences were observed in frequency of coded responses with respect to gender (F statistic 0.02, P-Value 0.88) or year of study the students were in (F statistic 1.13, P-Value 0.34). Differences were observed with respect to the university the respondent was from (F statistic 3.12 P-value 0.05), with this being most pronounced and significant for the differences in coding between MakCHS and MUST (F statistic 6.30 P-value 0.01). The codes that were generated from the analysis were further categorized into three themes based on Taylor and Hamdy's (2013) model of adult learning [17]: dissonance, consolidation and cognitive reorganisation for the other phases in the model that is, feedback organisation and refinement. These themes and the codes under each of them are described in more detail in the next paragraphs (Table 2). The theme on dissonance corresponds to the first stage in the model [17], in this stage the learner's existing knowledge is challenged and found to be incomplete. Several things that include the nature of the task, learner's motivation, stage of development and available resources affect the learner's engagement. The following codes and related recommendations were clustered under this theme: importance, time, relevance, teaching, content and course (see Table 2). The students responses coded under importance are described as the value students placed on learning about research methods. In addition to the statements in Table 1, respondents thought research methods were important for: future employment, motivating students to publish results, enhancing their ability to read and interpret publications, understanding research problems, and as a source of income. Interpretation of their own results from their studies and those they found in other peoples work is emphasized in this quotation: *“Sometimes we carry out research and fail to analyse or understand it because of very little or no information on biostatistics”*2^nd^ year Female Medical Radiology student.

**Table 1:** Distribution of coded responses according to participating university

Item	University			
	Makerere	Mbarara	Gulu	Total
Gender (N,% female)	342, 29.8	66, 39.4	18, 22.2	426
Year of study (mode, IQR*)	4, 3-4	3, 2-4	4 3-5	599
***Codes***				
Application	49	49	12	110
Content	23	5	-	28
Course	39	1	1	41
Importance	86	13	-	99
Mentorship	16	4	4	24
Program organization	18	1	2	21
Relevance	51	18	4	73
Teaching	36	6	2	44
Time	55	15	5	75
Total	373	112	30	515

* Inter-quartile range (IQR)

**Table 2:** Summary of the codes with examples of related text

Codes	No. (%)	Examples of statements
1. Application	110 (21.32)	“*Short period individual research projects to apply the theoretical knowledge-biostatistics is hard theoretically*” 5^th^ year male medical student “*Publishing research results could encourage and motivate us*” 4^th^ year pharmacy student “*I think that our teaching could be improved if we were given more opportunities to get hands on experience*” 2^nd^ year female dental student
2. Importance	99 (19.19)	“*It would help in improving research and helps medical students in interpreting statistics used in research*” 4^th^ year female nursing student “*It would help me make diagnosis & clinical impressions based on biostatistics of relevant diseases affecting specific regions, age groups and gender*” 4^th^ year medical student “*They would be practical in making Ugandan doctors to practice more evidence based medicine & less of text book based medicine, I think it is good but still lacking*” 3^rd^ year male medical radiology student “*More time should be allocated to teaching the course and different research articles should be got and interpreted to help give a better understanding*” 4^th^ year male pharmacy student
3. Time	76 (14.73)	e. “*Give it more time and attention and probably include as assessment “assessment drives learning*”” 4^th^ year medical student f. “*More time should be offered for research methods and biostatistics, online courses should be availed to students*” 5^th^ year male dental student g. “*More time should be given for the subject, emphasizing its importance and involving students in designing and carrying research*” 3^rd^ year medical student 3. “*Give it more time for students to appreciate it better should be taught early for example in year 1*” 4^th^ year medical student
4. Relevance	73 (14.15)	“*Training in research methods & biostatistics makes it easier for students to: carry out research, comprehensively find data, analyse & present & draw conclusions*” 2^nd^ year female medical student “*For proper interpretation of data for proper planning of health services*” 2^nd^ year male medical student “*Since its optional for those interested, I suggest they organize special sessions for those interested*” 3^rd^ year female medical student “*It could be more useful if it is made compulsory because some people never know what they want until it is forced on them*” 3^rd^ year female nursing student “*If it is taught extensively and applied in the day to day activities*” 4^th^ year male medical student
5. Teaching	44 (8.53)	“*If only they can be before lunchtime with more vibrant lecturers and a bit practical it would help*” 4^th^ year medical student “*The teaching should not be very stressing, my teaching seemed like a punishment*” 3^rd^ year female medical student “*Increase the number of lectures so that students can understand better*.” 4^th^ year pharmacy student“ “*It should be more involving and some tutorials should be organized at medical school because some tutors in the field did not know anything, more practical sessions needed*” 2^nd^ year pharmacy student
6. Courses	41 (7.95)	“*Isolate research methods & biostatistics as a course unit & then allocate 3 credit units, to it otherwise nothing will ever be achieved*” 4^th^ year medical student “*A separate short course of biostatistics should be given at some time during the course for all students to participate and should be mandatory*” 3^rd^ year male medical student “*By having a course unit on clinical biostatistics*” 3^rd^ year female medical student
7. Content	28 (5.43)	“*Teaching biostatistics as separate from community based education and service (COBES) (no guidance from field lectures- they have no clue). Teaching us statistical analysis including packages e.g SPSS*” 5^th^ year female medical student “*Should be done more often and the software should be made available to the students together with the books*” 3^rd^ year male medical student “*Evaluating the prevalence, morbidity and mortality rate*” 3^rd^ year male dental student
8. Mentorship	24 (4.65)	“*Involving the students practically in the research around campus, this would allow for proper grasping of the skills other than cramming*” 1^st^ year male medical student “*By allocating students to become research assistants of various experienced researchers*” 5^th^year male medical student
9. Organization of the program	21 (4.07)	“*Biostatistics learning should be gradual right from year 1 semester 1 and should be applied more prominently in COBES*” 2^nd^ year female pharmacy student “*If we did start studying using a research approach*” 3^rd^ year female nursing student “*How could it be of more use if it is non-existent, I do not understand the question!*” 5^th^ year male medical student

The next most frequently identified code in this theme was time with 76 codings (see Table 2). In this students voiced concerns with regards to the limited amount of time the content was being given yet to them it appeared important thus deserving more time. In some of the responses suggestions were made for ways to increase time, time on task, using: online courses, more practical sessions, making books available in addition to reworking the lecture schedules and class size (See in Table 2). The final aspect of time was the need for early exposure for maximal impact over the duration of the student's future professional life as seen in this quotation: *“Undergraduate teaching in research methods provides a unique opportunity to follow your interest in an area of research to focus on this for several years and make an impact”* 3^rd^ year Male Medical Radiology student. The items coded under relevance pointed to the students desire to understand why they were learning the research methods now. In summary the students thought the teaching of research methods would make the teaching of other subjects more relevant, promote the use of a common scientific language, impact the communities from which the students came from and make journal reading easier. Also under relevance the students wanted to see the societies they got the information from benefiting from the research findings as captured in this quotation: *“It could be more useful if the statistics obtained were used to improve health in the regions from which the statistics were obtained”* 2^nd^ year Male Medical Radiology student. The next code was on teaching with 44 codings. In this code student concerns on who was teaching the course were identified. The concerns ranged from the level of expertise of the teacher to how the teachers presented their material. Some of the students desired to learn the material under less stressful learning conditions for example in the morning as opposed to before lunch as seen in this quotation. *“If only they (lectures) can be before lunchtime with more vibrant lecturers and a bit practical it would help”* 4^th^ year medical student The next frequently cited code in this item was the courses with 41 codings. The students thought that the course material should be delivered as a stand-alone mandatory course with clear course content and examination leading to credits that appear on their transcript. Some of the student even suggested that a department should be created in the university to handle the teaching of research methods and biostatistics. The last code under dissonance was content with 28 codings. In this code student's suggestion for additional content in the form of exams, books available, software and scheduling were captured. The students also suggested that the exposure to this content should be repeated with increasing detail for deeper learning as they matured in their academic programs (see Table 2). The next theme, cognitive reorganisation, was used to lump together the codes under the other phases in the model that is, refinement, feedback, and organisation. According to the model, in each of these phases the students refine the new information into a series of concepts (refinement); then reorganise their knowledge into new schemas to make sense of the new information (organisation); and finally test this newly acquired knowledge against their peers and teachers (feedback) [17]. With the exception of the feedback stage the others occur in the students’ mental faculties thus the name of the theme, cognitive reorganisation. Under this theme were the codes application and mentorship. The code application captured students’ views on how they wanted to learn to apply the material they were being taught. It was the most frequently identified code with 110 codings (see Table 1 and Table 2). The opportunity to apply knowledge learned is a core feature of refinement and organisation of knowledge as captured in this quote. *“If we had data collection practically immediately after studying the theory and not wait for years”* 1^st^ year female medical student.

The next code group in this theme was mentorship with 24 codings. In mentorship students identified the need for opportunities for guided practical learning through involvement in departmental research, or engagement in the activities of different research organizations as a form of mentorship. Learning to do research in an area of interest to the mentor would result in greater mentor engagement in the learning process. This engagement is driven by the quality of feedback the mentor provides. The students linked the quality of feedback to the level of interest the mentor had in the area of research. *“Lecturers who understand that field”* 3^rd^ year male medical student The last theme was that corresponding to the consolidation phase of the adult learning model [17]. In this phase the learner reflects on what they have learned and the process they went through to learn. Under this were the one code capturing recommendations on organisation of the program with 21 codings. In this code the students expected the teaching of research methods and biostatistics to appear in all the courses in the respective programs. This would require the adoption of evidence-based teaching leading to a complete reorganization of the course. The students viewed this as a cross cutting learning activity as opposed to a single course as is summarized in this quotation: *“Biostatistics learning should be gradual right from year 1 semester 1 and should be applied more prominently in COBES”* 2^nd^ year female pharmacy student.

## Discussion

This study set out to explore students’ views on how to improve the teaching of research methods and biostatistics at three Ugandan public universities. The students who participated in the study appreciated the importance of learning research methods and biostatistics as a way of understanding research problems and appropriately interpreting statistical concepts during their training and post-qualification practice. They also identified the limited time allotted to teaching and inadequate educational resource materials as the main impediments to effective learning of research methods and biostatistics. They proposed several suggestions on ways to improve the teaching and learning of research methods and, ways of effectively utilizing the little time allotted to this course with a strong emphasis on putting what is learned into immediate practice as expected according to the principles of adult learning [15]. Health professional students have varied backgrounds and it is ever becoming more evident that traditional didactic approaches to teaching research methods and biostatistics neither engage student nor meet their demands [20]. In this study students voiced their discontent on the way research methods and biostatistics are taught with complaints ranging from the way the lectures are given to the timing of the sessions. Much of the teaching of statistics and research methodology is focused on refining the cognitive aspects (such as aptitudes and knowledge) students are expected to develop while ignoring the non-cognitive aspects (such as outlooks, perspectives, expectations and inspirations) [4]. Not addressing the student's expectations leaves the students very anxious which in turn results in poor engagement in the learning process by the student. Teaching statistics and research methods using the principles of adult learning, addresses the key concerns of the adult learner coming into a new learning situation. These include: (a) providing a comfortable and non-threatening learning environment, (b) course material is designed to meet learners’ needs, (c) supports the development and enhances learners’ self-esteem, (d) results in active and self-directed participation, (e) recognizes and makes use of learners’ past experiences, and (f) allows learners to monitor their own progress towards set targets/objective [15]. Aspects of each of these items are seen in each of the identified codes/quotations seen in this study. An example of the application of these adult learning principles is seen in the students request for more use of training methods that require active participation is enhance learning [15]. In this study students expressed the need for: opportunities to apply what they had learned, mentorship through guided practical learning and involvement in research activities with their teachers (see Table 1). Individuals learn attitudes, beliefs and behaviours through observing others [21]. Students do not have to learn the principles of research methodology from the lecture rooms alone; they also learn it from faculty by observation and working together on research projects and publications. Some authors report that most of the university teachers are themselves not conversant with all the statistical methods and rarely apply these approaches in their daily practice [22–24]. The result is that the students never get to see statistics being used as faculty are seen to insist of doing things the way they have always been done! Therefore universities should ensure that their faculty are well grounded in research methodology and also encourage them to involve their students in research and publication writing.

Another example of the students identifying adult approaches to learning is seen in suggestions to increase learning and developing durable skills in critical appraisal and statistical interpretation. Students proposed early and continuous exposure to research methods throughout the course of their academic programs. This they suggested should be complemented with; additional web based educational resource materials; and more hands-on practical teaching approaches such as case study interpretation, participation in research and publication writing. As students mature they become more independent and self-directed. Thus, they have to practice as this is the most effective way of teaching and mastering statistical skills [14]. Some authors have recommended a multi-media approach to teaching research methods and biostatistics by use of a combination of didactic lectures, small group tutorials, and videos and animations [25, 26]. Those who have gone ahead to implement this approach have reported positive results [20]. The analysis used in this study, informed by the adult learning model by Taylor and Hamdy's (2013) [17], helps to highlight the challenge students in these three public universities face with learning research methods. The observation that most of the recommendations were categorised within the dissonance phase of the learning model means that many of our learners fail to engage with the learning process and thus are not learning. From other studies on this subject we have noted that this could be a long-standing problem that has become institutionalised, as yesterday's students become today's faculty, thus perpetuating the cycle of dissonance [27]. This has implications for the participating institutions that will have to adjust their curricula and enhance their faculty development programs with respect to their various vision/mission statements [17] (Table 3). At the institutional level some of the activities that will reverse this trend include: encouraging productive student faculty research teams to foster feedback and mentorship; and, increase the time allotted to teaching research methods through the use of mandatory stand-alone examinable courses. These courses should be done repetitively with increasing depth for both faculty and students since it has been shown that statistical concepts and research methods are best learned and appreciated over time. This repetition allows the research methodology concepts to mature as students’ progress from basic to clinical disciplines [24]. This notion was clearly summarized by a second year pharmacy student in this quotation: *“Biostatistics learning should be gradual right from year 1 semester 1 and should be applied more prominently in COBES”*. Some of the shortcomings of this study included the use of inherently biased qualitative approach for the analysis of short open-ended responses to the probe question. Whereas the results of the analysis are very context specific, the number of respondents created an opportunity to do tallies that can be used as a rough indicator of where the institutions represented in the study lie with respect to the used adult learning model. Future studies may need to use more in-depth approaches involving more stakeholders. Despite the shortcomings the study findings are relevant at both the institutional and individual levels. For the student, it is important to foster student interest in research methods. The ease of understanding statistical and research concepts is directly dependent on the student's interest in the subject [28]. Since many students have negative views or tend to develop them towards research methods. Institutional interventions need to be put in place to change these negative views since these views can profoundly impact on the students learning and later application of research methodology concepts during the students training and later during their practicing careers [15].

**Table 3:** Vision and mission statements of the participating universities

University	Vision	Mission
**Makerere University College of Health Sciences**	To be the leading institution for academic excellence and innovations in Africa.	To provide innovative teaching, learning, research and services responsive to National and Global needs.
**Mbarara University for Science and Technology**	To be a centre of academic and professional excellence in Science and Technology.	To provide quality and relevant education at national and international level with particular emphasis on Science and Technology and its application to community development.
**Gulu University**	To be a pillar for academic, professional and sustainable development	To provide access to higher education, research and conduct quality professional training for the delivery of appropriate services directed towards community transformation and conservation of biodiversity

## Conclusion

This study has highlighted several challenges faced by students in public universities in learning research methods and biostatistics. In view of the large number of suggestions clustered under the dissonance phase of learning we conclude that the current teaching of research methods is not adequately engaging students in the learning process. This has resulted in the students’ failure to comprehend and appreciate the underlying principles governing the use of the various research methods. There is thus a need for the participating institutions to make individual learner and institutional adjustments to align the teaching of research methods with their vision and mission statements.

### What is known about this topic

- Health professional training curricula have increasingly become congested leading to the allocation of inadequate time to hands-on teaching of research methods and related research statistical approaches;
- Much of the university teaching on research methods focuses more on knowledge transfer, while giving little or no attention to context and application of the acquired knowledge;
- Students’ research outputs count greatly to research related status of most institutions of higher education, thus keeping track of student’ research related expectations and performance is a key quality control measure.

### What this study adds

- The current methods of teaching research methods leave most of the students in the dissonance phase of learning resulting in none or poor student engagement that results in a failure to comprehend and/or appreciate the principles governing the use of different research methods;
- Research methods and biostatistics should be delivered as a stand-alone mandatory course with clear course content and examination leading to credits that appear on academic transcript;
- Individual learners and academic institutions should make adjustments to align the teaching of research methods with their vision and mission statements.
